# Assessment of the Teratogenic Effect of Sulfadoxine-Pyrimethamine on the Chicken Embryo

**DOI:** 10.1155/2022/2995492

**Published:** 2022-03-15

**Authors:** Rachida Moussa Tari, Aboudoulatif Diallo, Emmanuelle Kouame, Phénix Assogba, Essotolom Badjabaissi, Lawson-Evi Povi, Batomayena Bakoma, Yao Potchoo, Kokou Tona

**Affiliations:** ^1^Department of Pharmaceutical Sciences, Faculty of Health Sciences, University of Lome, Lome, Togo; ^2^Faculty of Sciences, Laboratory of Poultry Science, University of Lome, BP 1515 Lome, Togo; ^3^Department of Animal Physiology and Pharmacology, Faculty of Sciences, University of Lome, Lome, Togo

## Abstract

**Background:**

The sulfadoxine-pyrimethamine combination is a product used in the intermittent preventive treatment (IPT) of malaria in pregnant women in our country. To date, there is very little data on the teratogenic effect of this product. This study proposed to evaluate the teratogenic effect of sulfadoxine-pyrimethamine on chicken embryos.

**Methods:**

The teratogenic effect of the product was evaluated on chicken embryos at a dose of 1.3 mg/g sulfadoxine and 0.06 mg/g pyrimethamine. The product was injected before the start of incubation and on days 12, 14, 16, and 18 of incubation. One batch received a double injection of the product on days 16 and 18 of incubation. The quality of the hatched chicks was evaluated by the Tona Score followed by the determination of hematological and biochemical parameters.

**Results:**

From the aforementioned, it appears that the eggs treated with sulfadoxine-pyrimethamine significantly decreased the hatchability rate of the eggs. The chicks obtained were all of very good quality. Apart from a significant decrease in the weight of the chicks of the batch that received the injection twice and a significant increase in the weight of the yolk sac of the chicks of the batch that received the injection on day 16 compared to the control, no variation was obtained. A significant increase in the white blood cell count of the chicks compared to the control was reported in the chicks of the batch injected before incubation and on day 12, as well as a significant increase in the platelet count of the chicks of the batch injected twice. For biochemical parameters, no significant difference was reported in ALT and AST.

**Conclusion:**

Sulfadoxine-pyrimethamine decreased egg hatch and caused an increase in embryo and chick mortality as well as a loss in relative chick weight and an increase in relative yolk sac weight. More in-depth studies would be needed on sulfadoxine-pyrimethamine teratogenicity and the benefit/risk ratio of this drug during pregnancy.

## 1. Introduction

Malaria is a parasitic disease caused by the genus *Plasmodium* transmitted to humans by the bites of infected female Anopheles mosquitoes. This disease remains one of the main causes of morbidity and mortality in the world and more particularly in Africa [1]. According to the World Health Organization (WHO), 229 million cases of malaria were reported with 409,000 deaths due to malaria in 2019 [2]. However, certain population groups are at increased risk of contracting malaria and developing the severe form of the disease. These include infants, children under 5 years of age, and pregnant women [3]. Several epidemiological data show that malaria is an endemic disease responsible for a high number of deaths, especially among children in West Africa [2]. Togo is one of the countries most affected by malaria in West Africa. In addition, maternal malaria is the most important risk factor for infant mortality [3]. Malaria in pregnancy is very harmful to the mother and the fetus and represents a public health concern.

To reduce the burden of malaria in pregnancy, WHO recommends a tripartite intervention package consisting of distribution and use of insecticide-treated nets, effective case management, and use of the association Sulfadoxine-Pyrimetamine (SP) for intermittent preventive treatment (IPT) of malaria in areas of moderate to severe transmission [4]. Thus, a regimen of at least three doses of Sulfadoxine-Pyrimetamine is recommended from the second trimester of pregnancy, from the 16th week of amenorrhea and at each scheduled prenatal visit until delivery. Each dose should be given at least 1 month apart [4]. The rate of use of Sulfadoxine-Pyrimetamine varies from nation to nation. For example, in Nigeria and Uganda, less than 30% of pregnant women received Sulfadoxine-Pyrimetamine [5]. In 2018, in Togo, the rate of pregnant women who received three doses of Sulfadoxine-Pyrimetamine during pregnancy was 45.50% [6]. However, few studies have evaluated the effect of Sulfadoxine-Pyrimetamine on the mother and child.

Furthermore, the use of certain drugs during pregnancy could involve risks for the embryo and/or the fetus. This could be the case of the sulfadoxine-pyrimethamine association. Indeed, sulfadoxine and pyrimethamine are inhibitors of the biosynthesis of tetrahydrofolate (THF) [7, 8]. By their inhibitory action of DHPS (dihydropteroate synthetase) for sulfadoxine [9] and DHFR (dihydrofolate reductase) for pyrimethamine [10], they thus induce folic acid deficiency. Folic acid (vitamin B9) from food and supplements is normally reduced to THF to be biologically active [11]. Moreover, folic acid deficiency in humans has been associated with an increased incidence of malformations [12]. In addition, some antifolate agents, including pyrimethamine, are known teratogens in animals, especially when used during the first trimester of pregnancy. Sulfonamides inhibit DHPS and sulfonamides other than sulfadoxine cause cleft palate and other malformations in rats and mice [13, 14]. Sulfonamides also have the ability to displace bilirubin from its binding sites inducing nuclear jaundice in the fetus, particularly with long half-life products such as sulfadoxine [15].

Based on previous data, the use of sulfadoxine-pyrimethamine combination could have adverse effects on the fetus. However, there are few relevant data on a possible malformative or fetotoxic effect of the sulfadoxine-pyrimethamine association when used during pregnancy [16]. It is within this framework that this study was conducted to produce scientific data on the teratogenic effect of this drug on the fetus by exploring tests on a new experimental model (the chicken embryo) used in many toxicity studies because it is easily manipulated [17]. This study aims to evaluate the teratogenic effect of the sulfadoxine-pyrimethamine combination in the chicken embryo to study embryonic and chick mortality, and to investigate possible malformations and toxicity in chicks.

## 2. Materiel and Methods

Our study was carried out in the laboratory of Poultry Production Techniques of the Regional Center of Excellence on Poultry Sciences (CERSA) and in the laboratory of Pharmacology and Toxicology of the Faculty of Health Sciences of the University of Lome, Togo.

### 2.1. Material

#### 2.1.1. Drug Substance

The drug substance used in this study was the combination of sulfadoxine (500 mg)/pyrimethamine (25 mg) (FANSIDAR^®^). This drug was purchased in a pharmaceutical dispensary in Lome and is used in the treatment of uncomplicated Plasmodium falciparum malaria and in IPT in pregnant women.

#### 2.1.2. Biological Material

The biological material used in this study consisted of Dutch Blue hatching eggs acquired from a Togolese producer.

#### 2.1.3. Equipment and Consumables

It was used during this study, an incubator (PAS REFORM^®^), electric sight, hatching trays, a centrifuge (HARAEUS Megafuge 1.0R), a hematology automat (MINDRAY BC 3000 Plus), and a biochemistry spectrophotometer (UV–1600 PC).

Five centiliter syringes and insulin syringes, precision balance, adhesive tape (Hypafix^®^), physiological water, hydrophilic cotton pads, EDTA, and dry tubes, pipettes, 100 uL and 1000 uL cones and others were used as consumables during this study.

## 3. Study Methods

### 3.1. Preparation of the Injectable Substance (Drug Substance)

The substance to be injected was prepared on the basis of the dosage of the sulfadoxine-pyrimethamine combination ratio to the average egg weight. Each tablet contains 500 mg of sulfadoxine and 25 mg of pyrimethamine; and the human dosage is 25 mg/kg of sulfadoxine and 1.25 mg/kg of pyrimethamine. The human dosage related to the average egg weight of 51.12 g resulted in a dosage of 1.3 mg/g sulfadoxine and 0.06 mg/g pyrimethamine of egg weight.

The mixture was then dissolved in 100 *μ*L of 0.9% physiological fluid (NaCl).

### 3.2. Evaluation of Possible Teratogenic Effects of Sulfadoxine-Pyrimethamine

#### 3.2.1. Purchase of Chicken Eggs and Manipulation

The Dutch Blue brand chicken eggs were purchased from a local producer in Lome (Togo). Eggs were selected that were no more than 24 hours old, medium in size, and visibly clean with no stains on the shell. Once in the laboratory, they were cleaned and placed on a scale. After weighing, the eggs were divided into batches (*n* = 50 eggs per batch) according to weight and then placed in the incubator under normal conditions (37.7°C; 55% relative humidity; 0.06% CO_2_; 1/60 min of turning). In the incubator, the largest part of the eggs (inner tube) is oriented upwards and the pointed end downwards. A total of eight (08) batches were formed. Prior to incubation, batches 2 and 3 were given physiological water and SP-medicated solution, respectively. At the 12th day of incubation, all eggs were mirrored and only the fertile eggs were used for the rest of the experiment. Nonfertile eggs were removed from the incubator and broken to determine the type of mortality: early or late mortality. Batches 4, 5, 6, 7, and 8 received SP solution (1.3 mg/0.06 mg) at day 12, day 14, day 16, day 16, and day 18 of incubation respectively. The batches formed were distributed as follows:

- Batch 1 (T): Control batch that did not receive anything.
- Batch 2 (NaCl): Control batch that received only 0.9% physiological water (NaCl).
- Batch 3 (SP_J0_): Batch that received SP before the start of incubation,
- Batch 4 (SP_J12_): Batch that received SP (1.3 mg/0.06 mg) on day 12 of incubation.
- Batch 5 (SP_J14_): Batch that received SP (1.3 mg/0.06 mg) on day 14 of incubation.
- Batch 6 (SP_J16_): Batch that received SP (1.3 mg/0.06 mg) on day 16 of incubation.
- Batch 7 (SP_J16-J18_): Batch that received SP (1.3 mg/0.06 mg) on 16 and 18 day of incubation.
- Batch 8 (SP_J18_): Batch that received SP (1.3 mg/0.06 mg) on day 18 of incubation.

After the injections, the pierced parts were closed with an adhesive tape (Hypafix^®^) and the eggs were again placed in the incubator with the same incubation conditions. At day 21, the machine was stopped and the chicks were taken out. When the machine was stopped, the unhatched eggs were counted and broken. This allowed the identification of clear eggs and those containing dead embryos. The hatching rate and mortality rate (delayed, early, and stillborn) were then determined.

### 3.3. Chick Quality at Hatching: Tona Score

The Tona score was done as previously reported by Tona et al. [17] with scoring activity, feathering and appearance, condition of eyes, conformation of legs, condition of navel area, remaining yolk sac, and status of the yolk membranes. It was expressed as a hedonic scale, and the quality score was calculated by summing up the scores for these characteristics. The score divides the chicks into groups of different qualities, with those scoring 100 being free of any abnormalities and being of the best quality [17].

### 3.4. Chick and Vital Organ Weights

After the evaluation of the quality of the chicks, six (06) chicks per batch were sacrificed, and then the heart, liver, and yolk sac were removed, weighed, and the relative weights of each isolated organ as well as that of the chick without yolk sac were calculated.

The following formulas were used to calculate the different parameters studied:

(1) $$\begin{array}{c} \text{Fertile hatching rates}=\frac{\text{Number of chicks at hatching}}{\text{Number of fertile eggs incubated}}\times 100,\\ \text{Morality rate}\left(\text{stillbirth}\right)=\frac{\text{Number of dead chicks}}{\text{Number of fertile eggs incubated}}\times 100,\\ \text{Relative weight of organs }\left(\text{liver, heart, yolk sac}\right)=\frac{\text{Organ weight}}{\text{Chick weight}}\times 100,\\ \text{Relative weight of chick without yolk sac}=\frac{\text{Chick weight without bag}}{\text{Chick weight}}\times 100. \end{array}$$

### 3.5. Hematological and Biochemical Examinations

Blood samples were taken in EDTA tubes and dry tubes for hematological and biochemical examinations. Hematological parameters determined were hemoglobin (Hb), red blood cell (RBC) count, white blood cell (WBC) count, platelets (PLT), hematocrit (Hte), mean corpuscular volume (MCV), mean corpuscular hemoglobin concentration (MCHC), and mean corpuscular hemoglobin content (MCHC). The determination of AST and ALT for biochemical examinations was performed.

### 3.6. Statistical Analysis of Data

GraphPad Prism 8 software was used to analyze our results. The results are expressed as mean values with the standard error of the mean (*m* ± SEM). Analysis of variance (ANOVA) was used to compare multiple groups. The difference between two groups was determined using the Tukey test. Differences were significant if the probability *p* was less than 0.05 (*p* < 0.05).

## 4. Results

### 4.1. General Observations

In our study, eggs treated with sulfadoxine-pyrimethamine showed reduced hatchability due to embryonic mortality, especially those of batches SPJ0, SPJ12, and SPJ16-J18. In the SPJ0 batch, there was a lot of early mortality embryos that died before day 7 of incubation with blackish deposits either at the bottom of the shell or above the allantois. We had no apparent malformations. Tona's score was 100 in all groups, meaning the chicks were of good quality.

### 4.2. Effect of Sulfadoxine-Pyrimethamine Combination on Hatching Rate

**Figure 1:**
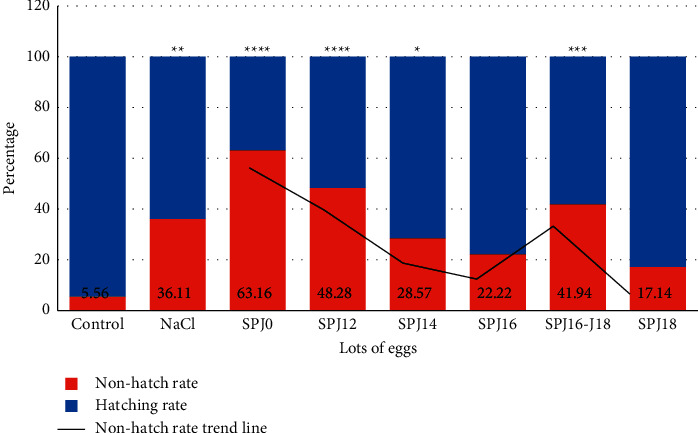
Nonhatching rate in different eggs batches. Eggs were incubated under normal conditions (37.7°C; 55% relative humidity; 0.06% CO_2_; 1/60 min of turning). Different treatments are made on different days. The nonhatching rate was then determined. Data are expressed as mean ± standard error of the mean (SEM) and analyzed by one-way ANOVA, followed by Tukey test (*p* < 0.05) as compared to the respective parameter value of control groups. ^∗^*P* < 0.05, ^∗∗^*P* < 0.01, ^∗∗∗^*P* < 0.001, ^∗∗∗∗^*P* < 0.0001 significant difference from control. ns = not significant. SP_J0_ = injection of sulfadoxine-pyrimethamine before the start of incubation; SP_J12_ = injection of sulfadoxine-pyrimethamine on day 12 of incubation; SP_J14_ = injection of sulfadoxine-pyrimethamine on day 14 of incubation; SP_J16_ = injection of sulfadoxine-pyrimethamine on day 16 of incubation; SP_J16-J18_ = injection of sulfadoxine-pyrimethamine on day 16 and day 18 of incubation; SP_J18_ = injection of sulfadoxine-pyrimethamine on day 18 of incubation.

The hatching and nonhatching rates of the different experimental batches and the control batch are presented in Figure 1. The results showed that the nonhatching rates in the NaCl, SPJ0, SPJ12, SPJ4, and SPJ16-J18 batches were significantly high compared to the control batch (*p* < 0.05) (Figure 1). A trend line shows a progressive decrease in the nonhatch rate from the days of product injection. The later the injection, the higher the hatching rate. On the contrary, in lot SPJ16-J18, where the injection was done twice, we note a strong increase in the nonhatching rate compared to lots SPJ16 and SPJ18. Each value represents the mean ± SEM.

### 4.3. Effect of Sulfadoxine-Pyrimethamine Combination on Stillbirth Rate

**Figure 2:**
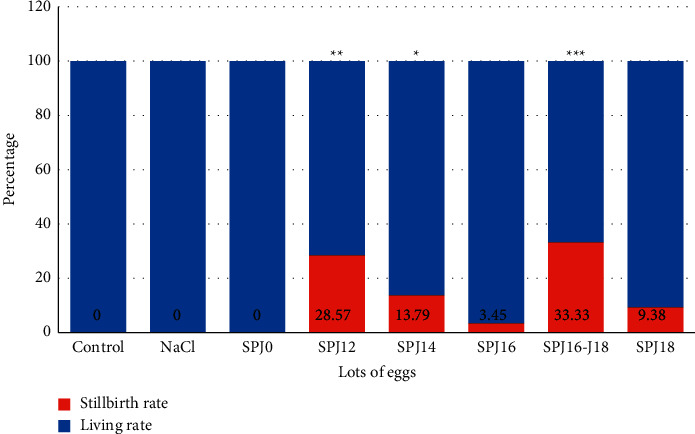
Stillbirth rate in the different eggs batches. Eggs were incubated under normal conditions (37.7°C; 55% relative humidity; 0.06% CO_2_; 1/60 min of turning). Different treatments are made on different days. The stillborn rate was then determined. Data are expressed as mean ± standard error of the mean (SEM) and analyzed by one-way ANOVA, followed by Tukey test (*p* < 0.05) as compared to the respective parameter value of control groups. ^*∗*^*p* < 0.05, ^*∗∗*^*p* < 0.01, ^*∗∗∗*^*p* < 0.001, ^*∗∗∗∗*^*p* < 0.0001 significant difference from control. ns = not significant. SP_J0_ = injection of sulfadoxine-pyrimethamine before the start of incubation; SP_J12_ = injection of sulfadoxine-pyrimethamine on day 12 of incubation; SP_J14_ = injection of sulfadoxine-pyrimethamine on day 14 of incubation; SP_J16_ = injection of sulfadoxine-pyrimethamine on day 16 of incubation; SP_J16-J18_ = injection of sulfadoxine-pyrimethamine on day 16 and day 18 of incubation; SP_J18_ = injection of sulfadoxine-pyrimethamine on day 18 of incubation.

Figure 2 represents the effect of sulfadoxine-pyrimethamine injection on the stillbirth rate. The results show that the stillbirth rates in the SPJ12, SPJ14, and SPJ16-J18 batches were significantly higher than the control batch (*p* < 0.05). These data are presented in Figure 2.

### 4.4. Effect of the Sulfadoxine-Pyrimethamine Combination on Chick Weight and Vital Organs

Table 1 represents the effect of sulfadoxine-pyrimethamine injection on the relative organ weights of chicks. The results show that sulfadoxine-pyrimethamine significantly decreased the weight of chicks in the SPJ16-J18 batch (*p* < 0.05) compared to the control batch. There was also a significant increase in yolk sac weight of chicks in the NaCl batch (*p* < 0.05) and the SPJ16 batch (*p* < 0.01) compared to the control.

### 4.5. Effect of the Sulfadoxine-Pyrimethamine Combination Used on Hematological and Biochemical Parameters

No significant difference was reported compared to the control in the biochemical parameters performed (AST, ALT) (Table 2). As for the hematological parameters, a significant increase in the white blood cell count of SPJ0 (*p* < 0.05) and SPJ12 (*p* < 0.01) lots compared to the control was reported (Table 3). There was also a significant increase (*p* < 0.01) in the platelet count of lot SPJ16-J18 compared to the control (Table 3).

## 5. Discussion

Our study was carried out on the chicken embryo and focused on the evaluation of the teratogenic effect of the combination sulfadoxine (1.3 mg/g)/pyrimethamine (0.06 mg/g) on Dutch Blue hen embryos.

In our study, treated eggs showed reduced hatchability due to embryonic mortality, especially those in the SPJ0, SPJ12, and SPJ16-J18 incubation batches compared to the control batches. The NaCl-treated batch had a higher nonhatchability rate than the control batch that received nothing. In the SPJ0 batch, there were many early mortalities, embryos that died before the 7th day of incubation with blackish deposits either at the bottom of the shell or above the allantois. These data could be explained by the fact that the injection of SP would have stopped embryonic development. Thus, these SP-induced mortalities are classified as early embryonic deaths. The rate of nonhatchability from SPJ0 to SPJ18 could be explained by the fact that the egg is an enclosed area where everything injected is metabolized and used. In addition, the heart, the first functional organ from the fourth or fifth day of incubation could be exposed to sulfadoxine-pyrimethamine. By this mechanism of embryonic development, it could be deduced that the early embryonic death obtained in this study would be due to the exposure of the heart to the administered product [18]. This gradual decrease in nonhatch rates observed in SPJ12, SPJ16, and SPJ18 batches would show embryo resistance to sulfadoxine-pyrimethamine. Late mortality due to a substance was also reported in 2015 by Saleemi et al. [19]. The same observations were made by Assogba et al. [18]. The latter have shown that from the 10th day of incubation, the embryonic development has evolved a lot and other organs are already in place and functional. Therefore, the fetus is more able to fight against toxic products capable of hindering its development. This suggests that the resistance of chick embryos to toxicants is age-related. The age-related increase in resistance of embryos to toxins is thought to be related to the activation of the detoxification mechanism when the liver and kidneys are functional according to Khan et al. [20]. The low hatching rate obtained in our study for batch 7 (SPJ16-J18) could be explained by a very high dose of sulfadoxine-pyrimethamine because of the reinjection on day 18 of incubation. The metabolism of the drug injected on day 16 would certainly not be completed before the injection on day 18. This would have pumped the heart and kidneys of the chicks, which would be the basis of the mortality rate. In addition, it is important to note that the NaCl-treated batch had a higher nonhatching rate than the control batch. This finding shows that the embryonic mortalities obtained in the SPJ0 batch would not only be due to the sulfadoxine-pyrimethamine evaluated but probably to other factors. High stillbirth rates in lots SPJ12, SPJ14, and SPJ16-J18 compared to the control lot were also reported. These results are similar to those of Nwachi [21] and Nwachi et al. [22] that reported 100% death of female embryos. Nevertheless, although we had no apparent malformations, the observed mortality could have been due to malformations that would probably have manifested themselves if the embryo had lived as Clark RL states in 2017 [23] in the Birth Defects Research. The latter reported that embryo deaths are sometimes the result of malformations and that it is possible that embryo deaths prevent the occurrence of malformations.

The chicks were of good quality after the Tona score. These results are similar to those of Philips Howard et al. [24] who worked on pregnant rats and rabbits and did not observe any malformations. It should be noted that according to the work of Tona et al. [17], the quality of the chicks can be related to the quality of the incubated eggs and the storage time of the eggs before incubation. Indeed, the storage of eggs before incubation can deteriorate the internal quality of the eggs, especially the height of the albumen which, during incubation, the albumin proteins move into the amniotic fluid and are swallowed by the embryo which are then either digested in the intestine or transferred to the yolk sac where they can be used after hatching. The same observations have been made by Assogba et al. [18]. The good quality of the chicks obtained in this study could be justified by the fact that this work was carried out on visibly clean 24-hour-old eggs without any spots on the shell.

Blood counts are also very important in toxicology studies. The hematopoietic system is one of the preferred targets of toxic substances and therefore an important parameter of human or animal physiology. Regarding the blood count (CBC), our study reported a significant (*p* < 0.05) increase in the number of white blood cells in SPJ0 and SPJ12 chicks compared to control chicks. These results are similar to those of a study conducted by Nwachi et al. [22]. The latter also showed an increase in neutrophil count in pregnant Wistar rats that received a therapeutic dose of intramuscular sulfadoxine-pyrimethamine on days 5, 12, and 19 of gestation. The hyperleukocytosis observed in our study could be explained by an inflammatory response of the body to sulfadoxine-pyrimethamine. However, injection of sulfadoxine-pyrimethamine had no significant effect on the red line. These results are contrary to those reported by Bakhiet et al. [25] who conducted a study on sulfadoxine-pyrimethamine taken orally in chicks. They reported a significant decrease in mean corpuscular hemoglobin volume, hemoglobin (Hb) level, and mean corpuscular hemoglobin content values compared to control chicks. These differences could be explained by the dose administered, the duration of the study, and the type of study design used. The measurement of certain biochemical parameters such as enzyme activities in tissues and body fluids plays a major role in the study of disease, diagnosis, and assessment of toxicity. The liver, kidneys, and lungs are the main organs affected by the metabolic reaction caused by toxic substances. Injection of sulfadoxine-pyrimethamine did not significantly affect the blood levels of AST or ALT. These data are contrary to those of Bakhiet et al. [25] who recorded significant increases in serum transaminase levels in the group of chicks treated with sulfadoxine-pyrimethamine. Biochemical changes of organ function biomarkers are linked to histological changes in the respective organ [26]. Since no biochemical changes were observed during this study, the histological examination was not performed.

In terms of weight, the injection of sulfadoxine-pyrimethamine in vivo did not have a significant effect on the weight of the chicks at hatching (*p* > 0.05) nor on the relative weight of the chicks except for that of the SPJ16-J18 batch, which was lower than that of the control batch (*p* < 0.05). These results are similar to those of Bakhiet et al. [25] who also had a decrease in the growth of the tested chicks after 2 weeks. Our results could be explained by the overdose of pyrimethamine because there was a double injection of sulfadoxine-pyrimethamine. The relative weights of the organs provide information on possible hypertrophy, atrophy, or swelling of these organs [27]. The injection of sulfadoxine-pyrimethamine had no significant effect on the relative weight of the weighed organs (heart and liver) except for the yolk sacs of chicks from the SPJ16 and NaCl batches, which were larger than the control batch. This could be explained by the fact that sulfadoxine-pyrimethamine and NaCl would each have caused a deficiency in linoleic acid, which would have slowed down the resorption of the yolk sac. Indeed, in the absence of linoleic acid, the yolk reserves are difficult to resorb.

## 6. Conclusion

In this study, we evaluated the teratogenic effect of the sulfadoxine-pyrimethamine combination on the chicken embryo. It was found that the injection of sulfadoxine-pyrimethamine into the egg resulted in a decrease of the hatchability, an increase of death of embryos and chicks as well as a loss of relative weight of chicks and an increase in the relative weight of the yolk sac. For hematological parameters, a significant increase in white blood cells and platelets was observed. It would be interesting to do an in-depth study of sulfadoxine-pyrimethamine teratogenicity which would confirm the benefit-risk ratio of this drug during pregnancy.

## Figures and Tables

**Table 1:** Effect of sulfadoxine-pyrimethamine injection on relative weights (%) of chick and organ.

Batch	Chicks	Chicks without yolk sac	Chicks with yolk sac	Heart	Liver
Control	31.53 ± 1.09	90.85 ± 1.02	3.91 ± 0.85	0.81 ± 0.07	2.85 ± 0.11
NaCl	35.88 ± 0.97	89.25 ± 1.58	7.61 ± 1.08^*∗*^	0.93 ± 0.07	2.71 ± 0.14
SP_J0_	28.00 ± 0.69	92.32 ± 0.94	4.60 ± 0.71	0.83 ± 0.07	3.10 ± 0.10
SP_J12_	33.78 ± 1.12	89.45 ± 1.81	6.61 ± 1.75	0.92 ± 0.06	3.06 ± 0.07
SP_J14_	34.18 ± 0.51	90.23 ± 1.13	6.44 ± 1.06	0.91 ± 0.03	3.11 ± 0.23
SP_J16_	32.86 ± 0.87	88.88 ± 2.14	7.94 ± 2.18^*∗∗*^	0.77 ± 0.06	2.75 ± 0.23
SP_J16-J18_	33.34 ± 0.58	87.30 ± 2.04^*∗*^	5.24 ± 0.44	0.75 ± 0.02	2.72 ± 0.08
SP_J18_	33.94 ± 0.74	93.42 ± 1.06	4.76 ± 0.59	0.75 ± 0.04	3.14 ± 0.19

Data are expressed as mean ± standard error of the mean (SEM) and analyzed by one-way ANOVA, followed by Tukey test (*p* < 0.05) as compared to the respective parameter value of control groups. SP_J0_ = injection of sulfadoxine-pyrimethamine before the start of incubation; SP_J12_ = injection of sulfadoxine-pyrimethamine on day 12 of incubation; SP_J14_ = injection of sulfadoxine-pyrimethamine on day 14 of incubation; SP_J16_ = injection of sulfadoxine-pyrimethamine on day 16 of incubation; SP_J16-J18_ = injection of sulfadoxine-pyrimethamine on day 16 and day 18 of incubation; SP_J18_ = injection of sulfadoxine-pyrimethamine on day 18 of incubation.

**Table 2:** Effect of injection of sulfadoxine-pyrimethamine combination on biochemical parameters (AST and ALT) of chicks.

Batch	AST (UI/L)	ALT (UI/L)
Control	2.02 ± 1.02	3.60 ± 2.59
NaCl	1.81 ± 1.14	1.24 ± 0.00
SP_J0_	3.42 ± 0.41	1.91 ± 1.70
SP_J12_	1.89 ± 1.37	2.50 ± 2.43
SP_J14_	1.45 ± 0.37	3.29 ± 0.38
SP_J16_	2.72 ± 1.13	3.07 ± 2.08
SP_J16-J18_	2.22 ± 1.11	3.31 ± 0.13
SP_J18_	2.80 ± 0.52	3.49 ± 1.19

Data are expressed as mean ± standard error of the mean (SEM) and analyzed by one-way ANOVA, followed by Tukey test (*p* < 0.05) as compared to the respective parameter value of control groups. SP_J0_ = injection of sulfadoxine-pyrimethamine before the start of incubation; SP_J12_ = injection of sulfadoxine-pyrimethamine on day 12 of incubation; SP_J14_ = injection of sulfadoxine-pyrimethamine on day 14 of incubation; SP_J16_ = injection of sulfadoxine-pyrimethamine on day 16 of incubation; SP_J16-J18_ = injection of sulfadoxine-pyrimethamine on day 16 and day 18 of incubation; SP_J18_ = injection of sulfadoxine-pyrimethamine on day 18 of incubation. ALT: alanine aminotransferase, AST: aspartate aminotransferase.

**Table 3:** Effect of sulfadoxine-pyrimethamine combination on hematological parameters of chicks.

Parameters	Control	NaCl	SP_J0_	SP_J12_	SP_J14_	SP_J16_	SP_J16-J18_	SP_J8_
WB (10^3^/l)	152.70 ± 3.24	160.35 ± 4.09	165.37 ± 1.75^*∗*^	168.25 ± 2.84^*∗∗*^	148.64 ± 5.14	160.20 ± 3.29	155.74 ± 6.30	158.20 ± 3.80
Hb (g/dL)	11.63 ± 0.75	12.72 ± 0.67	13.87 ± 0.18	12.85 ± 0.96	11.63 ± 0.54	13.13 ± 0.46	12.53 ± 0.88	13.25 ± 0.57
RB (10^6^/uL)	2.17 ± 0.14	2.38 ± 0.07	2.70 ± 0.05	2.30 ± 0.42	2.13 ± 0.09	2.45 ± 0.06	2.36 ± 0.13	2.62 ± 0.09
Hte (%)	31.72 ± 2.17	34.93 ± 1.46	38.88 ± 0.62	34.03 ± 4.82	30.90 ± 1.37	35.47 ± 0.87	33.78 ± 1.97	36.93 ± 1.34
MCV (fL)	146.10 ± 1.16	146.87 ± 2.93	144.32 ± 1.11	154.40 ± 11.16	145.45 ± 1.27	145.00 ± 1.49	143.02 ± 1.39	141.22 ± 1.47
MCH (pg)	53.60 ± 1.05	53.35 ± 1.94	51.38 ± 0.34	60.25 ± 12.64	54.70 ± 1.50	53.53 ± 0.98	52.75 ± 1.34	50.57 ± 1.04
MCHC (g/dL)	36.73 ± 10.78	36.28 ± 0.70	35.62 ± 0.25	39.48 ± 4.23	37.63 ± 0.82	38.62 ± 1.19	36.92 ± 0.72	35.78 ± 0.47
PLT 10³/UL	20.00 ± 3.81	20.20 ± 0.80	24.75 ± 3.25	25.33 ± 2.73	29.00 ± 2.92	16.00 ± 0.91	33.80 ± 2.22^*∗∗*^	26.75 ± 3.45

Data are expressed as mean ± standard error of the mean (SEM) and analyzed by one-way ANOVA, followed by Tukey test (*p* < 0.05) as compared to the respective parameter value of control groups. GB = white blood cells; NR = red blood cells; Hb = hemoglobin; Hte = hematocrit; MCV = average globular volume; MCH = average corpuscular hemoglobin content; MCHC = mean corpuscular hemoglobin concentration; PLT = platelets. SP_J0_ = injection of sulfadoxine-pyrimethamine before the start of incubation; SP_J12_ = injection of sulfadoxine-pyrimethamine on day 12 of incubation; SP_J14_ = injection of sulfadoxine-pyrimethamine on day 14 of incubation; SP_J16_ = injection of sulfadoxine-pyrimethamine on day 16 of incubation; SP_J16-J18_ = injection of sulfadoxine-pyrimethamine on day 16 and day 18 of incubation; SP_J18_ = injection of sulfadoxine-pyrimethamine on day 18 of incubation.

## Data Availability

All data generated or analyzed during this study are included in this published article.

## References

[1] Obiang P. C. N. (2021). Severe plasmodium falciparum malaria complicated by a symmetrical peripheral gangrene on an African child from an endemic area. *Health Sciences and Disease*.

[2] Léger Offono Enama M., Akono Ntonga P., Mbida Mbida A., Nopowo Takap N., Mbiada B., Etoile Ngo Hondt O. (2020). Le paludisme: connaissances, attitudes et pratiques des chefs de ménage de la region de l’ouest-Cameroun. *Journal of Applied Biosciences*.

[3] Tako E. A., Zhou A., Lohoue J., Leke R., Taylor D. W., Leke R. F. (2005). Risk factors for placental malaria and its effect on pregnancy outcome in Yaounde, Cameroon. *The American Journal of Tropical Medicine and Hygiene*.

[4] Rogerson S. J., Unger H. W. (2017). Prevention and control of malaria in pregnancy–new threats, new opportunities?. *Expert Review of Anti-infective Therapy*.

[5] https://www.mesvaccins.net/web/news/15000-rapport-mondial-sur-le-paludisme-en-2019-des-progres-a-soutenir-pour-l-elimination-du-paludisme.

[6] https://www.afro.who.int/sites/default/files/2019.09/RAPPORT%20ANNUEL%20DE%20PERFORMANCE%202018%20DU%20MSHP.pdf.

[7] Pietrzik K., Bailey L., Shane B. (2010). Folic acid and L-5-methyltetrahydrofolate. *Clinical Pharmacokinetics*.

[8] Nord F. F., Mull R. P. (2009). Recent progress in the biochemistry of Fusaria. *Advances in Enzymology and Related Areas of Molecular Biology*.

[9] Hitchings G. H. (1973). Mechanism of action of trimethoprim-sulfamethoxazole—I. *The Journal of Infectious Diseases*.

[10] Ferone R., Burchall J. J., Hitchings G. H. (1969). Plasmodium berghei dihydrofolate reductase isolation, properties, and inhibition by antifolates. *Molecular Pharmacology*.

[11] Goh Y. I., Koren G. (2008). Folic acid in pregnancy and fetal outcomes. *Journal of Obstetrics and Gynaecology*.

[12] Hibbard E. D., Smithells R. W. (1965). Folio acid metabolism and human embryopathy. *Lancet*.

[13] Peters P. J., Thigpen M. C., Parise M. E., Newman R. D. (2007). Safety and toxicity of sulfadoxine/pyrimethamine. *Drug Safety*.

[14] Clark R. L. (2017). Animal embryotoxicity studies of key non-artemisinin antimalarials and use in women in the first trimester. *Birth Defects Research*.

[15] Boitel E., Desoubeaux G. (2000). Antiparasitic treatments in pregnant women: update and recommendations. *Médecine et Maladies Infectieuses*.

[16] https://base-donnees-publique.medicaments.gouv.fr/affichageDoc.php?specid=61856062&typedoc=R#RcpSecuritePreclinique.

[17] Tona K., Bamelis F., De Ketelaere B. (2003). Effects of egg storage time on spread of hatch, chick quality, and chick juvenile growth. *Poultry Science*.

[18] Assogba P., Dougnon V., Hounsa E. (2021). Assessment of larval toxicity and the teratogenic effect of three medicinal plants used in the traditional treatment of urinary tract infections in Benin. *BioMed Research International*.

[19] Saleemi M. K., Khan M. Z., Khan A. (2015). Embryotoxic and histopathological investigations of in-ovo inoculation of aflatoxigenic fungal extracts in chicken embryos. *Pakistan Veterinary Journal*.

[20] Khan W. A., Khan M. Z., Khan A. (2014). Dietary vitamin E in White Leghorn layer breeder hens: a strategy to combat aflatoxin B1-induced damage. *Avian Pathology*.

[21] Uche-Nwachi E. O., Caxton-Martins A. E. (1998). Sulfadoxine-pyrimethamine embryopathy in Wistar rats. *Kaibogaku Zasshi Journal of Anatomy*.

[22] Uche-Nwachi E. O. (1998). Effect of intramuscular sulfadoxine-pyrimethamine on pregnant Wistar rats. *The Anatomical Record*.

[23] Clark R. L. (2017). Animal embryotoxicity studies of key non-artemisinin antimalarials and use in women in the first trimester. *Birth Defects Research*.

[24] Phillips-Howard P. A., Wood D. (1996). The safety of antimalarial drugs in pregnancy. *Drug Safety*.

[25] Bakhiet A. O., Abbaker M. E., Gadir W. S. A. (2006). Effect of dietary supplementation of aristolochia bracteolate and Astragalus gummiferor their combination on bovans-type chicks. *Journal of Animal and Veterinary Advances*.

[26] Shakil M. S., Hasan M. A., Uddin M. F. (2020). In vivo toxicity studies of chitosan-coated cobalt ferrite nanocomplex for its application as MRI contrast dye. *ACS Applied Bio Materials*.

[27] Amresh G., Singh P. N., Rao C. V. (2008). Toxicological screening of traditional medicine Laghupatha (Cissampelos pareira) in experimental animals. *Journal of Ethnopharmacology*.

